# Effects of Multispecies Probiotic Supplementation on Serum Bone Turnover Markers in Postmenopausal Women with Osteopenia: A Randomized, Double-Blind, Placebo-Controlled Trial

**DOI:** 10.3390/nu16030461

**Published:** 2024-02-05

**Authors:** Marut Vanitchanont, Sakda Arj-Ong Vallibhakara, Areepan Sophonsritsuk, Orawin Vallibhakara

**Affiliations:** 1 Reproductive Endocrinology and Infertility Unit, Department of Obstetrics and Gynaecology, Faculty of Medicine, Ramathibodi Hospital, Mahidol University, Bangkok 10400, Thailand; vanitchanontm@hotmail.com (M.V.); areepan.sop@mahidol.ac.th (A.S.); 2 Child Safety Promotion and Injury Prevention Research Center, Faculty of Medicine, Ramathibodi Hospital, Mahidol University, Bangkok 10400, Thailand; dr.sakda@gmail.com

**Keywords:** probiotics, multispecies probiotics, bone turnover markers, postmenopausal women, osteopenia, supplement, bone health, postmenopausal bone health

## Abstract

Probiotics have been found to have beneficial effects on bone metabolism. In this randomized, double-blind, placebo-controlled trial, the effects of multispecies probiotic supplementation on bone turnover markers were evaluated after 12 weeks. Forty postmenopausal women with osteopenia were included and randomly divided into two groups. The intervention group received multispecies probiotics, while the control group received identical placebo sachets daily. The baseline characteristics of both groups were similar. Still, the median serum bone resorption marker C-terminal telopeptide of type I collagen (CTX) was slightly higher in the multispecies probiotic group than in the placebo group (0.35 (0.12, 0.53) vs. 0.16 (0.06, 0.75); *p*-value = 0.004). After 12 weeks, the mean difference in serum CTX at baseline versus 12 weeks was significantly different between the multispecies probiotic and placebo groups (−0.06 (−0.29, 0.05) vs. 0.04 (−0.45, 0.67); *p*-value < 0.001). The multispecies probiotic group showed a significant decrease in serum CTX at 12 weeks compared with baseline (*p*-value 0.026). However, the placebo group showed no significant change in serum CTX (*p*-value 0.18). In conclusion, multispecies probiotics may have a preventive effect on bone through their antiresorptive effect in osteopenic postmenopausal women.

## 1. Introduction

The adult skeleton comprises the cortical (80%) and trabecular (20%) bones distributed across different bone sites. Bone is considered a dynamic tissue that constantly goes through remodeling via communication between cellular components, including cells of the osteoblast lineage (osteoblasts and osteocytes) and cells involved in bone resorption (osteoclasts). These cells collectively form the bone remodeling units (BRUs), which are active throughout life and maintain bone homeostasis; up to 4 million BRUs are stimulated yearly, and approximately 1 million BRUs are actively participating in remodeling at any particular time. The functions of BRUs are controlled by various local and systemic factors, such as inflammatory cytokines, hormones, transcription, and growth factors, via different extrinsic and intrinsic pathways. Examples of cytokines related to bone remodeling include tumor necrotic factor α (TNF-α), interleukin-1 (IL-1), interleukin-6 (IL-6), and prostaglandin E2 (PGE2). In addition, B-lymphocytes, T-lymphocytes, and dendritic cells may also be involved in remodeling either directly or indirectly through the secretion of specific factors or cytokines [1,2,3].

Postmenopausal women are defined as those remaining amenorrheic for at least 12 consecutive months due to permanent cessation of ovarian function. As women approach the menopausal period, progressive decline in female reproductive hormones may be associated with signs and symptoms such as hot flushes, mood swings, insomnia, and vulvovaginal atrophy, with varying degrees of severity for each individual. Furthermore, long-term consequences from this aging process, such as cardiovascular diseases, cognitive loss, osteoporosis, and cancer, may be experienced [4,5]. Considering bone integrity, estrogen deficiency leads to greater resorption activities than formation. The decline in estrogen levels throughout the aging process leads to bone loss via different mechanisms. Immunohistochemical studies reveal that the levels of estrogen receptor α (ER-α) and estrogen receptor β (ER-β) in cells involved in bone remodeling, including those within the BRUs, T-lymphocytes, and monocytes, are reduced. However, in normal circumstances, estrogen exerts its effects via canonical Wnt/β-catenin, stimulating osteoblasts in bone formation. Furthermore, receptor activators of nuclear factor kappa-B ligand (RANKL) and osteoprotegerin (OPG) pathways are simultaneously activated, which act through osteoclasts to reduce bone resorption. Premenopausal women are, therefore, expected to undergo balanced bone remodeling regarding formation and resorption to maintain bone homeostasis [6,7,8]. Recent molecular studies have found that estrogen may also exert its immunomodulatory effects either directly by modulating the secretion of specific cytokines or indirectly via the Fas/Fas ligand (FasL) signaling pathway, a well-known pathway involved in apoptosis [9]. As estrogen levels become deficient, the first stage of accelerated trabecular and cortical bone loss becomes apparent due to increased activation and decreased apoptosis of osteoclasts. This precedes the second stage with a slower rate of life-long bone loss as osteoblasts are continuously downregulated [10,11]. Another consequence of estrogen deficiency is the uprising of inflammatory processes due to the secretion of proinflammatory and osteogenic cytokines such as IL-1, IL-6, IL-7, TNF-α, RANKL from osteoblasts, and activated B-lymphocytes and T-lymphocytes [12,13,14].

Interestingly, estrogen deficiency alters the equilibrium of gut microbiota and corresponding disease pathways, such as increased risks of obesity, insulin resistance, type 2 diabetes, fatty liver disease, cardiovascular diseases, and inflammatory bowel disease [15,16,17]. Furthermore, defective bone metabolic functions and various bone diseases may also be apparent. Previous studies have focused on existing mechanisms of how the intestinal tract can influence bone health. Firstly, it regulates the absorption of minerals essential for healthy bone, such as calcium, magnesium, and phosphorus [18]. Secondly, various endocrine factors, including gut-derived factors such as serotonin and incretins, may affect the absorption of minerals and influence bone remodeling by modulating inflammatory responses. Incretins, such as glucose-dependent insulinotropic polypeptide (GIP) and glucagon-like peptide-1 (GLP-1), are released minutes after nutrient ingestion and help dispose of ingested nutrients quickly. GIP and GLP-1 activate receptors on islet β-cells, leading to glucose-dependent insulin secretion, induction of β-cell proliferation, and enhanced resistance to apoptosis. GIP and GLP-1 also play a role in maintaining bone health and promoting bone formation by stimulating osteoblast proliferation and inhibiting apoptosis [19]. Furthermore, mutations in the Lrp5 gene, which is widely expressed as a Wnt coreceptor and inhibits the expression of Tph1, the rate-limiting biosynthetic enzyme for serotonin in enterochromaffin cells of the duodenum, affect bone formation by inhibiting serotonin synthesis and osteoblast proliferation [20].

Over the past decade, many studies in animal models have been performed to explore the association between gut microbiota and bone health [21,22]. Among postmenopausal women, Rettedal EA reported the significantly different taxonomic compositions of gut microbiomes between healthy, osteopenic, and osteoporotic participants classified based on the T-score of bone mineral density. The study involved 86 participants whose body composition, bone density, and fecal metagenomes were analyzed [23]. Based on previous reports, it can be inferred that the influences of intestinal microbiota on bone undoubtedly involve complex processes and may be time-dependent. In contrast, the role of probiotic supplementation in regulating bone health is much stronger, with a greater number of studies depicting its beneficial effects.

Probiotics are dietary supplements containing live nonpathogenic microorganisms, including different genera of bacteria such as Lactobacillus, Bifidobacterium, Bacillus, Escherichia, Enterococcus, and yeasts such as *Saccharomyces*. Although naturally found in mucous membranes such as the oral cavity, skin, urinary and genital organs, and intestines, probiotics are commonly seen in dietary supplements and fermented products (e.g., milk products, beer, meat, and kimchi). Administration of adequate probiotics may confer health benefits to the human host regarding treating and preventing certain pathological conditions [24,25,26,27]. Various mechanisms of action of probiotics concerning the beneficial effects on human health, including bone, have been proposed based on in vitro and in vivo studies. Firstly, they may regulate intestinal functions by strengthening the integrity of the epithelial barrier, expressing more tight junction proteins, and reducing antigen transfer and subsequent stimulation of intestinal immune cells. Secondly, a reduction in proinflammatory cytokines, pro-osteoclastogenic cytokines, and oxidative stress was also demonstrated. Thirdly, bacteria themselves can synthesize numerous vitamins and enzymes necessary for matrix and bone growth, such as vitamins D, K, C, and folate. There may be many more mechanisms involved that remain to be investigated. These are likely complex, as multiple components with varying proportions of microorganisms may act through distinct and overlapping pathways within the host [28,29,30,31,32,33,34].

Therefore, this study aims to evaluate the changes in the standard serum bone resorption marker CTX and bone formation marker P1NP in postmenopausal women with osteopenia after supplementation with multispecies probiotics for three months. The study is conducted based on the hypothesis that probiotics could reduce bone resorption through decreased differentiation and functions of osteoclasts and, hence, serum CTX. Thus, this study explores the potential for multispecies probiotic supplements as an adjunct or an alternative in preventing postmenopausal osteoporosis.

## 2. Materials and Methods

### 2.1. Study Design

The study protocol was developed based on the Consolidated Standards of Reporting Trials (CONSORT) declaration. A double-blinded, randomized, placebo-controlled trial was conducted at the Department of Obstetrics and Gynecology, Faculty of Medicine, Ramathibodi Hospital, Bangkok, Thailand. This study aimed to evaluate the effects of multispecies probiotics on standard serum bone turnover markers, including bone resorption marker C-terminal telopeptide of type 1 collagen (CTX) and bone formation marker N-terminal propeptide of type 1 procollagen (P1NP) after 12 weeks of supplementation. The primary outcome was to evaluate the change in bone resorption marker CTX and bone formation marker P1NP in serum. The secondary outcome was to assess any adverse events. 

### 2.2. Participants

A study was conducted at the Gynecologic Outpatient Unit and Menopause Clinic, Department of Obstetrics and Gynecology, Faculty of Medicine, Ramathibodi Hospital, Bangkok, Thailand, between 1 March and 30 September 2023. The study included postmenopausal women with osteopenia aged between 45 and 70 years. Postmenopausal women were defined as those who have had amenorrhea for at least 12 consecutive months, undergone bilateral oophorectomy, or have a measurement of serum follicle-stimulating hormone (FSH) > 40 IU per liter. The diagnosis of osteopenia was made by having a bone mineral density measured by Dual-energy X-ray Absorptiometry T-score of between −1 and −2.5 at the lumbar spine, femoral neck, or total hip. All participants signed the informed consent form. Women with a body mass index (BMI) of <18 or >35 kg/m^2^, a history of fragility fracture, metabolic bone diseases, or chronic diseases potentially involving bone (e.g., severe renal or liver diseases, diabetes mellitus, thyroid or parathyroid diseases, autoimmune diseases, and vitamin D deficiency or insufficiency), cancer, or malabsorptive or eating disorders were all excluded. Also excluded were those who were taking medications potentially affecting bone metabolism (e.g., menopausal hormone therapy, bisphosphonates, raloxifene, calcitonin, growth hormone, parathyroid hormones, and steroids), anticoagulants, and medications or supplements containing probiotics within three months before the start of the study.

### 2.3. Sample Size Calculation

Sample size calculation in this study was based on the difference in serum bone turnover marker CTX in the randomized, double-blind, controlled trial by Jafarnejad S et al. using version 1.4.1 of n4Studies for comparing continuous outcomes [35]. Based on this reference study, the mean CTX and standard deviation for the treatment group were 0.43 and 0.02, respectively. For the control group, the mean CTX and standard deviation were 0.45 and 0.02, respectively. Considering the type I error of 5% (α = 0.05) and type II error of 20% (β = 0.2; power = 80%), 17 participants were required in each study group. After accommodating a data loss of 20%, 40 postmenopausal women were included in the study.

### 2.4. Randomization, Blinding, and the Study Protocol

All participants were assigned to either study group at a 1:1 ratio using computer-generated blocks of four randomization sequences. A trial identification number was used to identify each participant, and the assignment of treatment code was performed. Participants and investigators were all blinded to the group allocation. However, the pharmacists who prepared the sachets were exposed to the allocation. Participants in the intervention group received one sachet of multispecies probiotics per day, which could be taken orally before any meal for 12 weeks. Each sachet taken by the intervention group contains *Lactobacillus reuteri* GL-104 1.5 Billion CFU, *Lactobacillus paracasei* MP-137 0.6 Billion CFU, *Lactobacillus rhamnosus* MP108 0.6 Billion CFU, *Lactobacillus rhamnosus* F-1 0.3 Billion CFU, *Lactobacillus rhamnosus* BV77 0.6 Billion CFU, *Bifidobacterium animalis* ssp. *lactis* CP-9 2.4 Billion CFU, *Bifidobacterium longum* ssp. *longum* OLP-01 1 Billion CFU, *Bacillus coagulans* 1 Billion CFU, and 270 milligrams of inulin (prebiotics) (CMED PRODUCTS 1994 Company Limited, Bangkok, Thailand). In contrast, participants allocated to the placebo group received sachets produced with the same size, shape, odor, color, and packaging as the probiotic sachets but contained only 270 milligrams of inulin. Similar to the intervention group, participants could take one sachet per day orally before any meal. Participants in both groups received at least 1200 milligrams of calcium daily and 20,000 IU of vitamin D2 per week. Although they were asked to avoid food, beverages, or supplements containing probiotics, such as yogurt and kimchi, during the study, usual medications for their underlying diseases could still be taken. In addition, they were encouraged to perform routine daily activities, including their lifestyle, diet, and exercise.

### 2.5. Data Collection and Measurements

Baseline characteristics of the participants were collected at the time of enrollment, including age, menopausal age and type, parity, history of smoking and alcoholic drinking, underlying diseases, current medications, and exercise status. A physical examination was also performed, including body weight, height, body mass index, and blood pressure measurements. Attention was given to abnormal findings from history taking and physical examination suggesting other secondary causes of bone loss with further laboratory investigations undertaken. Baseline bone mineral density at the lumbar spine, femoral neck, and total hip were also recorded for all participants.

As byproducts of bone remodeling, bone turnover markers have been shown to possess clinical usefulness. They aid in stratifying risks for fragility fracture and help monitor response and adherence to treatment. Compared with BMD, these markers collected from urine or serum are often used in clinical trials as they change more rapidly. The International Osteoporosis Foundation (IOF) and the International Federation of Clinical Chemistry and Laboratory Medicine (IFCC) Working Group on Bone Marker Standards (WG-BMS) have evaluated the clinical potential of bone turnover markers (BTMs) in predicting fracture risk and monitoring treatment. Based on their recommendations, the serum bone resorption marker C-terminal telopeptide (CTX) and bone formation marker N-terminal propeptide of type 1 procollagen (P1NP) were used. Serum CTX is bone-specific, and its detection results from the breakdown of bone collagen through cleavage of cross-linked type I collagen by osteoclastic enzyme cathepsin K during resorption. In healthy individuals, the production of CTX in serum has a circadian rhythm with its peak in the second half of the night and nadir in the late afternoon. Unsurprisingly, it has been shown to decrease during treatment with antiresorptive medications. In contrast, P1NP is a trimeric peptide comprising two type 1 procollagen-α1 chains and a procollagen-α2 chain, which are noncovalently bonded. During bone formation, the propeptide extensions at the amino- and carboxy-terminals are cleaved off from the procollagen type I molecule and released into the bloodstream. The collagen molecule is subsequently deposited to form the osteoid matrix, enabling serum P1NP to represent histomorphometric measures of bone formation. Unlike serum CTX, it has very low circadian variations and increases during treatment with bone-forming therapies [36,37,38,39,40]. These were measured using an automated Cobas E602 chemiluminescence immunoassay (Roche Diagnostic, Mannheim, Germany). Intra-assay coefficients of variation for serum CTX and P1NP were 1.6% (0.004 ng/mL) and 1.7% (0.524 ng/mL), respectively. Inter-assay coefficients of variation for serum CTX and P1NP were 2.2% (0.006 ng/mL) and 2.6% (0.797 ng/mL), respectively. Each participant’s fasting venous blood sample was collected at 7–10 a.m. on the enrollment day and after 12 weeks to minimize variations.

Compliance with the consumption of sachets and any adverse effects were monitored monthly through phone interviews. After 12 weeks, all participants visited the hospital and brought the allocation package with the remaining sachets for investigators to check compliance. Participants who consumed at least 80 percent of the sachets provided at the beginning of the study were considered to have good adherence. Participants were also asked whether they regularly consumed calcium and vitamin D2 by subjectively recalling their missed days. Any minor or significant adverse events were explored and recorded through interviewing by the investigators with the participants.

### 2.6. Statistical Analysis

The ‘participants’ baseline characteristics are reported descriptively. According to the Shapiro–Wilk test, continuous variables were tested for normality. Normally distributed data were presented as mean ± standard deviation (SD), while non-normally distributed data were shown as median (range). Discrete variables were reported in counts (percentages). Statistical analyses were performed using STATA Version 15.0 (College Station, TX, USA). A comparison of the continuous variables in parametric data was performed using the Student *t*-test. However, a comparison of the continuous variables in nonparametric data was undertaken using the Mann–Whitney U test. A paired *t*-test and the Mann–Whitney U test for normal and non-normal distribution data were used to identify any within-group differences when comparing baseline and final serum bone turnover markers. Pearson’s chi-squared or Fisher’s exact test was used for categorical data. A type I error (α) of 0.05 was considered statistically significant. Analysis using the intention-to-treat method was performed in this study.

### 2.7. Ethical Approval

This study was approved by the Human Research Ethics Committee, Faculty of Medicine, Ramathibodi Hospital, Mahidol University (MURA2023/1999, approval date: 20 January 2023). Moreover, the study protocol was submitted to the Thai Clinical Trials Registry; TCTR (www.thaiclinicaltrials.org, accessed on 24 March 2023) with clinical trial registration number TCTR20230326002.

## 3. Results

### 3.1. Protocol Flow Diagram

**Figure 1:**
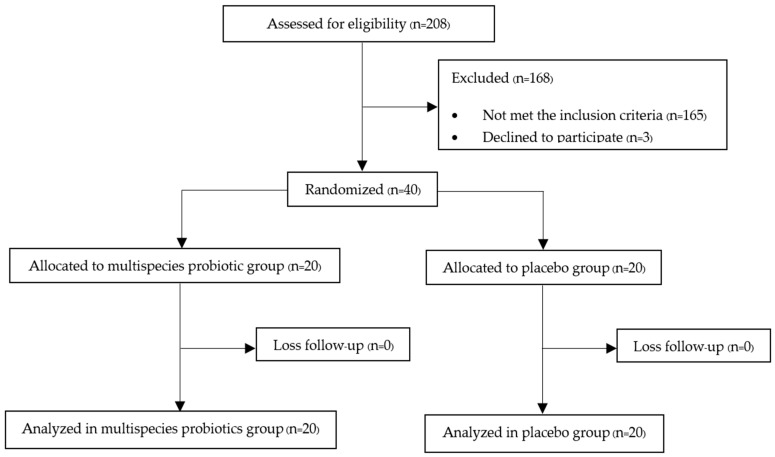
Protocol flow diagram of the study.

The protocol flow diagram is shown in Figure 1. Two hundred and eight postmenopausal women were assessed for eligibility, and 40 participants were finally included in our study. These participants were randomly and equally assigned into either the multispecies probiotic or placebo group and completed the study by the end of the twelfth week. There were no losses to follow-up in the study, possibly due to the follow-up strategy conducted by the authors in regularly contacting the participants in both groups once a month to monitor compliance and any adverse events. Fortunately, all participants in both the multispecies probiotic and placebo groups had good compliance, according to the number of remaining sachets counted on the day of the final follow-up visit. In addition, they were also compliant in consuming calcium and vitamin D2 supplements administered since the beginning of the study.

### 3.2. Serum Bone Turnover Markers

The baseline characteristics of the participants in both the multispecies probiotic and placebo groups are shown in Table 1. There were no significant differences in the baseline characteristics between the two groups. The majority of the participants in both groups had at least one underlying disease, which was mostly well-controlled dyslipidemia and hypertension. It is important to note that the participants with poorly controlled underlying diseases or those that could affect bone metabolism were excluded from the study. Three participants in the placebo group had a history of occasional alcoholic drinking, but they were all classified as light drinkers as none consumed greater than three times a month. All participants in both groups had no history of smoking. Concerning exercise, most participants in both groups had a history of exercise, which was low to moderate in intensity.

**Figure 2:**
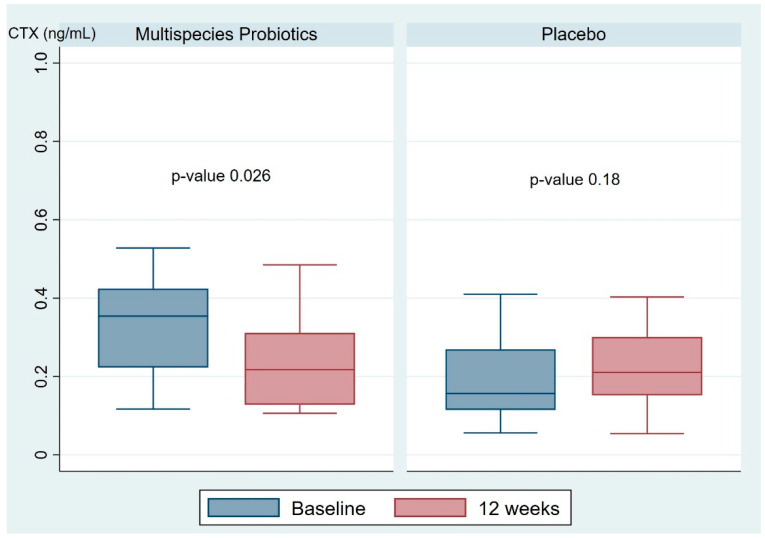
The box plot represents the changes in serum bone resorption marker CTX in the multispecies probiotic and placebo groups. CTX; C-terminal telopeptide of type I collagen.

The primary outcome of this study was to evaluate the change in serum CTX and P1NP after three months of intervention. As shown in Table 2, there was a significant difference in serum CTX between the two groups at baseline (0.33 (0.12, 0.53) in the multispecies probiotic group and 0.23 (0.56, 0.75) in the placebo group; *p*-value 0.004). However, the mean difference in the serum bone resorption marker CTX at baseline versus 12 weeks was significantly different between the multispecies probiotic and placebo groups (−0.06 (−0.29, 0.05) and 0.04 (−0.45, 0.67), respectively; *p*-value < 0.001). However, no significant difference in mean difference of serum bone formation marker P1NP between the multispecies probiotic and placebo groups was observed (−2.69 (−27.47, 10.59) and 2.38 (−32.15, 18.78), respectively; *p*-value 0.06). Furthermore, as shown in Figure 2, the multispecies probiotic group showed a significant decrease in serum CTX at 12 weeks compared with baseline (*p*-value 0.026). However, no significant change in serum CTX was demonstrated in the placebo group. Regarding serum P1NP, there were no significant changes at 12 weeks compared with baseline in both the multispecies probiotic group (*p*-value 0.64) and placebo group (*p*-value 0.86).

### 3.3. Adverse Events

Four participants in the placebo group and two in the multispecies probiotic group reported adverse reactions during the study period. However, the two groups had no significant difference in these adverse reactions (*p*-value 0.66). All self-reported adverse reactions in both groups were mild and self-limiting disturbances in bowel habits, which spontaneously resolved within days to a few weeks after taking the intervention assigned to them. The examiners recorded these data during the monthly phone interviews with all participants. At the hospital’s final follow-up visit at 12 weeks, none of the participants reported any further adverse events.

## 4. Discussion

Our study is a randomized, double-blind, placebo-controlled trial, which evaluated the effects of multispecies probiotic supplementation for three months on the recommended standard serum bone turnover markers CTX and P1NP in postmenopausal women with osteopenia. The probiotics used in our study comprised *Lactobacillus reuteri*, *Lactobacillus paracasei*, *Lactobacillus rhamnosus*, *Lactobacillus rhamnosus*, *Lactobacillus rhamnosus*, *Bifidobacterium animalis* ssp. *lactis*, *Bifidobacterium longum* ssp. *longum*, *Bacillus coagulans*, and 270 milligrams of inulin (prebiotics). In the probiotic group, a significant decrease in serum bone resorption marker CTX was observed as a significant difference between the mean difference in serum bone resorption marker CTX at baseline versus 12 weeks between the multispecies probiotic and placebo groups (−0.06 (−0.29, 0.05) and 0.04 (−0.45, 0.67), respectively; *p*-value < 0.001). However, we did not find a significant mean difference in serum bone formation marker P1NP between the groups. This depicts the protective benefits of multispecies probiotic supplementation against postmenopausal bone loss. In line with previous studies in estrogen-deficiency-induced animals and postmenopausal women, the effect of probiotics in slowing down osteoclast-induced bone resorption is demonstrated [21,35]. Based on other studies, probiotic treatment may also decrease inflammatory mediators and cytokine levels in the gut and bone marrow. These changes consequently send signals to stem cells, osteoblasts, and osteoclasts to affect bone homeostasis [35,41,42,43,44,45,46,47]. Endocrine factors produced by the gut, such as incretins and serotonin, may also influence bone cells [19,20]. Thus, the reduction in proinflammatory and osteolytic cytokines may alter the expression of anti-osteoclastogenic cytokines, leading to downregulation of osteoclast formation and bone resorption. However, these effects of probiotics are beyond the scope of our current research.

Estrogen deficiency and aging are physiologically linked, in which both are associated with postmenopausal osteoporosis. The lack of estrogen can alter the expression of estrogen target genes, increasing the secretion of IL-1, IL-6, and tumor necrosis factor (TNF). During menopause, there is a decrease in estrogen, which can cause an increase in bone resorption markers. The menopausal transition is a critical time for bone health because there is a rapid loss of bone mass and strength within three years after the last menstrual period. In addition to the decrease in bone mass, there are changes in bone macrostructure and microarchitecture that can be measured using composite strength indices and indices of trabecular thickness and connectivity [48,49]. Secondly, the aging process causes various bone changes that affect their structure and metabolism, increasing the risk of osteoporosis and fractures. The trabecular and cortical bone, including the marrow cellularity, undergo alterations at a histological level. Mesenchymal stem cells switch to follow an adipogenic fate, increasing adipose tissue within the bone marrow. Moreover, a build-up of senescent cells in bone tissue produces a senescence-associated secretory phenotype. This process involves gradual impairments of regenerative mechanisms, leading to a decline in the functionalities of tissues such as bone. Additionally, aging can affect the fate of bone cells, leading to an increased rate of osteoclast and osteoblast apoptosis, affecting bone resorption and formation. In fact, the rate of bone resorption increases with age, leading to bone loss and fragility. An example of age-related change in bone structure is the increase in periosteal diameter, which can weaken bones without a decrease in total bone mass. Elderly individuals may also experience more difficulty in absorbing calcium, leading to calcium deficiency and secondary hyperparathyroidism. These contribute to bone resorption and decreased bone mass [50,51].

Probiotics have been found to positively affect bone health through the anti-osteoclastogenic effect in maintaining intestinal barrier integrity and preventing toxins from entering systemic circulation, causing inflammation. Zyrek and colleagues have shown that the administration of probiotics containing Escherichia coli Nissle 1917 in enteropathogenic *E. coli*-induced gut dysbiosis increased claudin expression and impeded increases in intestinal permeability [52]. Recently, the ability to enhance the absorption and availability of minerals such as calcium, selenium, zinc, magnesium, and potassium by some strains of probiotics has also been gaining attention. The benefits of probiotics on bone depend on specific strains, strain–mineral affinity, dosage and duration of administration, and individual differences among hosts. Examples of absorption enhancement mechanisms include regulation of tight junctions between gut epithelial cells, alterations of the gut’s pH, increased expression of proteins essential for cellular transport, and production of short-chain fatty acids (SCFAs) such as acetate and propionate. SCFAs may exhibit anti-inflammatory properties, increase cecal villi surface area, and aid in paracellular transport pathways. Through increased availability of minerals and nutrients, probiotics may improve BMD and lower fracture risks in fracture-prone populations such as postmenopausal women [53,54,55,56,57].

Our findings indicate that twelve-week multispecies probiotic supplementation can impede bone resorption in postmenopausal women with osteopenia. Many animal studies have demonstrated osteoprotective effects on bone. Administration of different strains of probiotics, such as Lactobacillus and Bifidobacterium, in hormone-deficient and ovariectomized (OVX) mice has been shown to restore bone loss effectively. Chiang SS and Pan TM studied the effects of soy skim milk fermented with *Lactobacillus paracasei* and *Lactobacillus plantarum* on OVX mice. The study lasted eight weeks and showed that supplementation increased levels of aglycone isoflavones, soluble calcium, and vitamin D3. Moreover, the treatment group had higher trabecular bone volumes and numbers compared with the OVX and sham-OVX control groups [41]. Another study by Britton et al. found that four-week treatment with *Lactobacillus reuteri* ATCC PTA 6475 significantly protected bone loss in OVX mice via decreases in osteoclast bone resorption markers, including activators tartrate-resistant acid phosphatase 5 (TRAP5) and RANKL. Interestingly, the OVX-induced increases in bone marrow CD4+ T-lymphocytes, which promote osteoclastogenesis, were also suppressed [58]. Moreover, Li J-Y et al.’s study also supported the beneficial effects of Lactobacillus treatment in preventing trabecular bone loss due to estrogen deficiency by reducing gut permeability and dampening intestinal and basement membrane inflammation. They used a model of OVX in specific-pathogen-free mice and ovarian sex steroid inhibitor (leuprolide acetate) in germ-free mice treated with either *Lactobacillus rhamnosus* GG (LGG) or a probiotic supplement VSL#3 (containing four species of *Lactobacillus*, three species of *Bifidobacterium*, and *Streptococcus thermophiles*) for four weeks. The study showed that LGG and VSL#3 significantly prevented the decrease in femoral bone density and trabecular thickness and number compared with the controls. However, supplementation with a nonprobiotic strain of *Escherichia coli* or a mutant LGG did not provide this protection. The increased serum bone resorption marker CTX found in germ-free mice with ovarian suppression from leuprolide acetate compared with those receiving LGG was similar to our study. In our study, serum CTX was increased in the placebo group after twelve-week supplementation, although there was no statistical significance. These findings demonstrate that the increased gut permeability may trigger inflammatory pathways crucial for inducing bone loss in sex-steroid-deficient mice [21]. To further elucidate the benefits of Bifidobacterium treatment, Parvaneh et al. demonstrated that *Bifidobacterium longus* treatment for 16 weeks in OVX rats resulted in increases in bone density, trabecular number and thickness, and femoral strength. Compared with the sham group, treatment modulated osteoclast formation and activity by preventing OVX-induced osteoclast increase over the femur’s bone surface [42].

There are currently few well-designed randomized controlled trials that have been conducted to explore the effects of probiotics on bone in postmenopausal women. The outcomes for some of these studies include the standard serum bone resorption marker CTX, which is the marker of interest in our study. Jansson et al. studied 249 healthy postmenopausal women with a T-score greater than −2.5. The women were randomly divided into two groups and given either a probiotic supplement (containing *Lactobacillus paracasei DSM 13434*, *Lactobacillus plantarum DSM 15312*, and *Lactobacillus plantarum DSM 15313*) with 1 × 10^10^ CFU per capsule or a placebo for twelve months. The results showed a significant decrease in lumbar spine bone mineral density (BMD) in the placebo group, while the probiotic group had negligible bone loss. However, there were no differences between the groups in BMD at the total hip, trochanter, and femoral neck, bone resorption markers (serum CTX and urine N-terminal telopeptide/creatinine), bone formation markers (serum procollagen type I N-terminal propeptide [P1NP] and osteocalcin), and proinflammatory markers (TNF-α and high-sensitivity C-reactive protein (hs-CRP)). This implies that probiotic treatment with three *Lactobacillus* strains for twelve months in nonosteoporotic, healthy, early-postmenopausal women significantly reduced loss of lumbar spine BMD compared with placebo [44]. A further study by Nilsson et al. demonstrated the reduced loss of tibia total volumetric BMD in osteopenic postmenopausal women treated with twelve months of *Lactobacillus reuteri* 6475 compared with those receiving placebo [45]. In 2018, Takimoto T and colleagues conducted a study in Japan that involved examining the treatment effect of the probiotic *Bacillus subtilis* C-3102 (C-3102) for six months on bone mineral density (BMD) and its influence on gut microbiota in healthy postmenopausal Japanese women. The results showed that in the probiotic group, at twelve weeks, there was a decrease in the bone resorption marker tartrate-resistant acid phosphatase isoform 5b (TRACP-5b), and at twenty-four weeks, there was a significant increase in hip BMD when compared with the placebo group. In addition, the study showed that the gut microbiome was modulated by the treatment, as there were significant increases in the relative abundance of Bifidobacterium after twelve weeks and decreases in Fusobacterium after twelve and twenty-four weeks of treatment [46]. The effects of probiotic supplementation containing seven bacterial species (*Lactobacillus casei* 1.3 × 10^10^ CFU, *Bifidobacterium longum* 5 × 10^10^ CFU, *Lactobacillus acidophilus* 1.5 × 10^10^, *Lactobacillus rhamnosus* 3.5 × 10^9^, *Lactobacillus bulgaricus* 2.5 × 10^8^ CFU, *Bifidobacterium breve* 1 × 10^10^ CFU, and *Streptococcus thermophilus* 1.5 × 10^8^ CFU per 500 milligrams) for six months in osteopenic postmenopausal women were also evaluated by Jafarnejad and his team. This was a randomized, double-blind, placebo-controlled trial in which participants in both groups were given 500 milligrams (mg) of calcium and 200 international units (IU) per day. A significant decrease in the serum bone turnover markers CTX, bone-specific alkaline phosphatase (BALP), TNF-α, and parathyroid hormone (PTH) in the group that received probiotics compared with the placebo group was seen. However, no significant difference was observed between the two groups regarding bone mineral density (BMD) at the lumbar spine and total hip [35]. The decrease in serum CTX is in line with our study, suggesting the antiresorptive action of multispecies probiotic treatment. A recent meta-analysis by Jiawei Y and colleagues gathered data from five randomized controlled trials involving 497 postmenopausal women. The study found that the lumbar spine bone mineral density (BMD) was significantly higher in the group that received probiotics than the control group. Additionally, the probiotic group had significantly lower serum CTX levels than the control group. However, subgroup meta-analyses showed no significant differences between the groups’ serum BALP, osteocalcin, OPG, and TNF-α levels [47]. These results align with our study’s findings in further supporting probiotics’ antiresorptive and anti-inflammatory roles on bone in an estrogen-deprived state.

Despite the existence of effective postmenopausal osteoporosis treatments to minimize fracture risk, the absolute number of fractures is most remarkable in women who have not developed osteoporosis [59]. Currently, management of osteopenia in postmenopausal women includes adequate calcium and vitamin D supplements and nonpharmacologic strategies such as a balanced diet, weight-bearing exercise, prevention of falls, and avoidance of risk factors of bone loss. Although some antiresorptive medications, including hormonal therapies, are recommended for preventive use in postmenopausal women with high risk for fractures, their adverse effects often impact adherence and treatment success rates [60]. This underpins the necessity for exploring alternative treatments that are safe, inexpensive, and able to prevent postmenopausal bone loss leading to fractures effectively. The current study shows that combining multispecies probiotic supplementation with adequate calcium and vitamin D can effectively exert an antiresorptive effect on the bone of osteopenic postmenopausal women. In addition, this alternative seems safe as there was no significant adverse effect. Bowel disturbances were experienced by some patients at initiation of use in both groups, which were mild and self-limiting. Evidence has shown that the long-term use of probiotics enables the restoration of a healthy gut microbiome and improvements in symptoms of bowel disorders such as irritable bowel syndrome and ulcerative colitis [61,62].

The strength of this study is the randomized, double-blind, placebo-controlled trial study design with an intention-to-treat analysis. Another strength is that the probiotic supplement used in our study combines different genera species, which have previously been investigated and proved to benefit bone in animals and humans. In addition, all participants in our study groups had good compliance (≥80%) concerning medication intake, including the intervention, calcium, and vitamin D supplements. We believe this is due to our monthly phone interviews with all participants to monitor compliance and adverse effects closely. Some limitations should be considered when interpreting the findings of our study. First, baseline serum CTX levels in the probiotic group were higher than in the placebo group. Therefore, participants in the probiotic group had more room for change, and the actual effect of probiotics in decreasing this serum bone resorption marker may be questioned. Although randomization intended to have similar baseline CTX levels between the study groups, the situation in our study could have happened by chance. Still, there was a significant difference in the mean difference of serum CTX at two time points between the groups. Subsequent studies with larger sample sizes may eliminate this difference and enhance the results of our study. Secondly, the follow-up duration was insufficient to detect a significant change in bone formation marker (P1NP) and bone mineral density. Also, the slightly small sample of participants might have affected our results. Thirdly, we did not evaluate all recommended baseline laboratory investigations for secondary bone loss just before the study initiation, including serum vitamin D levels. Due to the nature of the study design, we believe that the intention of randomization allowed for a similar distribution of participants with varying vitamin D levels between the study groups. In addition, an equal dosage of vitamin D supplement was given to all participants in both groups from the initiation of the study. However, thorough history taking and physical examination were performed on all participants, with further evaluation of specific laboratory investigations if necessary. In addition, most participants in both groups already had some components of laboratory results available during their past visits. Indeed, this is a preliminary study for future research with a larger sample of patients and a longer duration of follow-up. The results of future studies may enhance our findings, allowing for the possibility of probiotics to be recommended as an alternative or add-on treatment in preventing postmenopausal osteoporosis.

## 5. Conclusions

Postmenopausal women are inevitably at risk for progressive bone loss and eventually osteoporosis due to estrogen deficiency and the aging process. Our study has demonstrated that multispecies probiotic supplementation for 12 weeks in osteopenic postmenopausal women may retard the increase in serum bone resorption marker CTX by downregulating osteoclast-induced bone resorption without significant adverse effects. Further experimental research may support our findings and prove its clinical usefulness before considering it as an alternative or add-on modality for the management of osteopenia in the postmenopausal period.

## Figures and Tables

**Table 1:** Baseline characteristics of the participants.

Characteristics	Multispecies Probiotic (n = 20)	Placebo (n = 20)
Age (years) ^a^	62 ± 5.07	64.05 ± 3.58
Age at menopause (years) ^a^	49.10 ± 4.99	50.10 ± 4.20
Menopausal type ^b^ Natural Surgical	16 (80%) 4 (20%)	16 (80%) 4 (20%)
Body mass index (kg/m^2^) ^a^	23.35 ± 3.77	24.20 ± 2.78
Parity ^b^ Nulliparous Multiparous	8 (40%) 12 (60%)	5 (25%) 15 (75%)
Underlying diseases ^b^ Yes No	16 (80%) 4 (20%)	17 (85%) 3 (15%)
Alcoholic drinking ^b^ Yes No	0 20 (100%)	3 (15%) 17 (85%)
Exercise time ^b^ No exercise <150 min/week ≥150 min/week	4 (20%) 15 (75%) 1 (5%)	8 (40%) 11 (55%) 1 (5%)
Baseline bone mineral density (g/cm^2^) ^a^ Lumbar spine Femur neck Total hip	0.86 ± 0.09 0.66 ± 0.08 0.79 ± 0.08	0.86 ± 0.11 0.64 ± 0.05 0.79 ± 0.06

Notes: ^a^ data are expressed as mean ± standard deviation (SD); ^b^ data are expressed as numbers (percentage).

**Table 2:** Comparison of serum bone turnover markers between the multispecies probiotic and placebo groups at baseline and after 12 weeks.

Serum Bone Turnover Marker	Multispecies Probiotic (n = 20)	Placebo (n = 20)	*p*-Value
CTX (ng/mL) Baseline ^a^ 12 weeks ^a^ Mean difference ^a^	0.33 (0.12, 0.53) 0.24 (0.11, 0.49) −0.06 (−0.29, 0.05)	0.23 (0.56, 0.75) 0.26 (0.54, 1.00) 0.04 (−0.45, 0.67)	0.004 * 0.90 <0.001 *
P1NP (ng/mL) Baseline ^b^ 12 weeks ^b^ Mean difference ^a^	55.45 ± 19.30 52.69 ± 17.99 −2.69 (−27.47, 10.59)	55.07 ± 28.53 56.60 ± 27.92 2.38 (−32.15, 18.78)	0.96 0.60 0.06

Notes: ^a^ data expressed as median (range), ^b^ data expressed as mean ± standard deviation (SD). CTX, C-terminal telopeptide of type I collagen; P1NP, N-terminal propeptide of type I procollagen. * *p*-value < 0.05 assigned as being statistically significant.

## Data Availability

Data are available on request due to restrictions on privacy or ethics. The data presented in this study are available on request from the corresponding author. The data are not publicly available due to the ethics and rights of the Faculty of Medicine, Ramathibodi Hospital, Mahidol University.
